# Diet and Nutrition in IBD—Progress and Gaps

**DOI:** 10.3390/nu11081740

**Published:** 2019-07-27

**Authors:** Maitreyi Raman, Subrata Ghosh

**Affiliations:** 1 Division of Gastroenterology, University of Calgary, 6D33 TRW Building, 3280 Hospital Drive NW, Calgary, AB T2N 4N1, Canada; 2 Institute of Translational Medicine, NIHR Biomedical Research Centre, University of Birmingham, Birmingham B15 2TT, UK

The incidence and prevalence of Inflammatory Bowel Disease (IBD) has rapidly increased worldwide and now is a global disease with some of the highest rates observed in North America [1]. IBD is believed to arise from a shared interaction between genetic and environmental influences, resulting in an imbalance between pro-inflammatory and anti-inflammatory signaling, however the etiology of this imbalance and of IBD itself is incompletely understood [2]. Environmental risk factors for both disease onset and risk for disease exacerbation are attractive to explore, as these may be modifiable, and have the potential to represent a therapeutic opportunity with few adverse side effects. Patients identify associations between diet and disease flare and the topic of diet and IBD is among the frequently asked questions by patients to their gastroenterologist. However, guidelines for dietary recommendations as primary therapy are lacking [3]. Additionally, IBD is associated with malnutrition, sarcopenia, and micronutrient deficiencies, all of which can reduce quality of life, increase morbidity, and contribute to symptoms [4,5]. The recently published literature assessing relationships between diet, nutrition, and IBD has increased substantially in the past 2 years, reflecting the urgency to find therapeutic solutions to meet patient needs and reduce disease related complications.

Specifically, epidemiologic data often implicate dietary lipids and fatty acids imbalance, in addition to animal proteins, as increasing predisposition to IBD, while vitamin D and fiber intake may be protective [6]. Some of these are dietary components are traditionally considered part of a healthy diet, such as vegetables, fruits, dietary fibers, n-3 polyunsaturated fatty acids (n-3 PUFA), n-9 PUFA, and vitamin D, while others, such as meats, n-6 PUFA, and refined sugars are often considered part of the ‘unhealthy’ Western diet, but it is unclear to what extent any of these are specific for IBD. Considerable evidence has emerged that diet targets innate and adaptive immunity in the intestinal barrier and gut microbiome in addition to correcting ‘malnutrition’ inherent in IBD [7,8]. With the tremendous amount of research in the gut microbiome in IBD, it is inevitable that interest in many aspects of diet and nutrition in IBD will increase exponentially, as highlighted in this issue. However, it is imperative that studying nutritional intervention in IBD evolves new paradigms and does not simply follow drug development paths. Intervening in early disease and in IBD sufferers in remission to prevent relapse is especially attractive.

This Special Issue of Nutrients, “Nutrition in Inflammatory Bowel Disease”, includes a collection of 21 manuscripts that richly describe the vibrant and diverse state of nutrition research in IBD. Close to half of the contributions are review articles that provide perspectives on mechanisms through which diet could play an important role in the pathogenesis of IBD, while another important review summarizes the state of randomized clinical trials in Ulcerative Colitis (UC), highlighting gaps and areas for research [9]. The high number of review articles on the topic of dietary mechanisms and IBD truly reflects the acute interest in this growing field. The stark lack of clinical trials exploring dietary relationships and gut inflammation is also very revealing and is a further call to action for IBD researchers.

Eight original articles, ranging from in vitro to animal models and human clinical research, explore various nutrition-focused themes that include probiotics, symbiotics, vitamin D, dietary intake and composition, protein dosing, obesity, myopenia, and exclusive enteral nutrition on diverse IBD outcomes [4,10,11,12,13,14,15,16].

Vidal-Lletjos et al. identified that moderately high protein dosing modulated mucosal healing, however high the protein intake, had deleterious consequences in animal models [14]. These results are undoubtedly of interest for developing nutrition prescriptions in the clinical setting and require further study in human populations. Taylor et al. described suboptimal dietary intake and low adherence with Mediterranean diet guidelines as not keeping with anti-inflammatory dietary patterns [4]. Shinde et al. demonstrated that symbiotic supplementation ameliorated disease activity index and histological scores in animal models, warranting human trials [12]. Of interest, Bryant et al. determined that, over 24 months of longitudinal follow-up, the body composition of patients with IBD changed, with gains of fat mass but decreases in lean muscle mass [16]. The morbidity that may be associated with these findings deserves further attention.

These valuable manuscripts contribute toward advancing knowledge about current dietary intake and composition in patients with IBD, describe existing nutritional deficiencies including myopenia, and provide a platform of nutritional supplements with biologic plausibility to be subjected to further trials.

We believe this compendium is a useful summary of the nutrition progress in IBD. The need for further nutrition intervention trials with diets that are mechanistically sensible and address the industrialization of food remains as a loud unspoken statement.
